# Prevalence of adhesin and toxin genes in *E. coli* strains isolated from diarrheic and non-diarrheic pigs from smallholder herds in northern and eastern Uganda

**DOI:** 10.1186/s12866-016-0796-2

**Published:** 2016-08-05

**Authors:** Kokas Ikwap, Jenny Larsson, Magdalena Jacobson, David Okello Owiny, George William Nasinyama, Immaculate Nabukenya, Sigbrit Mattsson, Anna Aspan, Joseph Erume

**Affiliations:** 1 College of Veterinary Medicine, Animal Resources and Biosecurity, Makerere University, P. O. Box 7062, Kampala, Uganda; 2 Faculty of Veterinary Medicine and Animal Science, Swedish University of Agricultural Sciences, P.O. Box 7070, SE-750 07, Uppsala, Sweden; 3 National Veterinary Institute, Uppsala, 751 89 Sweden

**Keywords:** AIDA-I, F4, *Escherichia coli*, Hemolytic, Piglets

## Abstract

**Background:**

Enterotoxigenic *E. coli* (ETEC) significantly contribute to diarrhea in piglets and weaners. The smallholder pig producers in Uganda identified diarrhea as one of the major problems especially in piglets. The aim of this study was to; i) characterize the virulence factors of *E. coli* strains isolated from diarrheic and non-diarrheic suckling piglets and weaners from smallholder herds in northern and eastern Uganda and ii) identify and describe the post-mortem picture of ETEC infection in severely diarrheic piglets. Rectal swab samples were collected from 83 piglets and weaners in 20 herds and isolated *E. coli* were characterized by PCR, serotyping and hemolysis.

**Results:**

The *E. coli* strains carried genes for the heat stable toxins STa, STb and EAST1 and adhesins F4 and AIDA-I. The genes for the heat labile toxin LT and adhesins F5, F6, F18 and F41 were not detected in any of the *E. coli* isolates. Where the serogroup could be identified, *E. coli* isolates from the same diarrheic pig belonged to the same serogroup. The prevalence of EAST1, STb, Stx2e, STa, AIDA-I, and F4 in the *E. coli* isolates from suckling piglets and weaners (diarrheic and non-diarrheic combined) was 29, 26.5, 2.4, 1.2, 16, and 8.4 %, respectively. However the prevalence of F4 and AIDA-I in *E. coli* from diarrheic suckling piglets alone was 22.2 and 20 %, respectively. There was no significant difference in the prevalence of the individual virulence factors in *E. coli* from the diarrheic and non-diarrheic pigs (*p* > 0.05). The main ETEC strains isolated from diarrheic and non-diarrheic pigs included F4/STb/EAST1 (7.2 %), F4/STb (1.2 %), AIDA/STb/EAST1 (8 %) and AIDA/STb (8 %). At post-mortem, two diarrheic suckling piglets carrying ETEC showed intact intestinal villi, enterocytes and brush border but with a layer of cells attached to the brush border, suggestive of ETEC infections.

**Conclusion:**

This study has shown that the F4 fimbriae is the most predominant in *E. coli* from diarrheic piglets in the study area and therefore an F4-based vaccine should be considered one of the preventive measures for controlling ETEC infections in the piglets in northern and eastern Uganda.

**Electronic supplementary material:**

The online version of this article (doi:10.1186/s12866-016-0796-2) contains supplementary material, which is available to authorized users.

## Background

Diarrhea is a major clinical manifestation of many diseases in livestock [1]. In pigs, diarrheal diseases are of economic concern particularly in piglets and weaners due to mortality, treatment costs, loss of weight and growth retardation in survivors [2–4]. Enterotoxigenic *Escherichia coli* (ETEC) are among the major causes of diarrhea in piglets and weaners [5].

The severity of ETEC infection depends on many factors, including the strain of the ETEC, age and health status of the host, stress, environmental and dietary factors [2, 6–9]. In particular, the aetiology of ETEC diarrhea in weaned pigs, called post-weaning diarrhea [10], is complex with ETEC being one of the critical factors [11].

The ETEC contribute to or cause diarrhea by first adhering to host receptors in the brush border of enterocytes in the duodenum, jejunum and /or ileum using adhesins [12], and secondly by producing toxins that when absorbed, cause efflux of water and electrolytes into the intestinal lumen and /or reduced intestinal absorption [13–15]. This is seen as diarrhea, resulting in dehydration, acidosis and death with minimal or no structural alteration of the intestinal mucosa [16, 17]. The ETEC adhesins are fimbrial or non-fimbrial proteins on the cell membrane encoded by genes located either on virulence plasmids or on the bacterial chromosome [18, 19]. Adhesins that have been known for a long time to be associated with ETEC from pigs are F4, F5, F6, F18 and F41 [17, 20]. Recently, another *E. coli* adhesin called “adhesin involved in diffuse adherence”, (AIDA), was found to be associated with diarrhea in piglets [21]. In 2007, another non-fimbrial adhesion called porcine attaching and effacing-associated factor (paa) that was originally identified in enteropathogenic *E. coli* strains was suggested to play a big role in the pathogenesis of ETEC infections [22] and recently, paa was reported to be associated with F4-positive ETEC from diarrheic piglets [23]. Longus (CS21), a type IV pilus of ETEC has also been reported to mediate adherence to pig intestinal epithelial cells and contribute to pathogenesis in mice [24]. In addition, the F1-like fimbriae have been demonstrated in ETEC isolates from diarrheic piglets that lacked other fimbriae [25]. However, the role played by the F1 fimbriae in disease is still debatable since they are also found in commensal bacteria. Further, other studies on diarrheic piglets suggest the occurrence of yet-to-be identified adhesins [16]. Some of the ETEC toxins expressed during bacterial adherence are plasmid-regulated and include the heat stable toxins STa and STb, heat labile toxin I (LT I) and *E. coli* aggregative heat stable toxin 1, EAST1 [26, 27]. Recently, Jobling and Holmes isolated *E. coli* from diarrheic and non-diarrheic animals carrying the chromosomal genes for the LTII toxins with further analysis suggesting that the LTII genes were prophage-encoded [28]. However, the contribution of EAST1 to diarrhea in piglets is in doubt [21, 29]. One ETEC strain can carry genes for one or more of the adhesins and toxins. Knowledge about prevalent adhesins has been employed to prepare anti-adhesin vaccines for control of ETEC infections through the vaccination of sows before parturition, thus enabling the piglets to acquire passive immunity through colostrum [30, 31].

In Uganda, the majority of pigs are kept by smallholder farmers many of whom frequently experience losses due to diarrhea in their piggeries. Diarrhea in piglets attributed to ETEC infections has been suspected to occur, however, no attempt has been made to confirm and identify ETEC strains involved. This study was carried out to; i) isolate and characterize the ETEC strains from diarrheic and non-diarrheic piglets and weaners from smallholder herds in northern and eastern Uganda with at least one diarrheic piglet or weaner and ii) identify and describe the post-mortem picture attributable to ETEC in severely diarrheic piglets. This study reported isolation of ETEC strains and presence of ETEC diarrhea in piglets and /or weaners from smallholder herds.

## Methods

### Study area and design

This was a cross-sectional study carried out from 2011 to 2014 in Gulu and Soroti districts, located in northern and eastern Uganda, respectively. The location of Gulu district is between longitude 30° 21' east to longitude 32° east and latitude 2° north to latitude 4° north. The location of Soroti district is between longitude 30° 01' east and longitude 34° 18' east and latitude 1°33' north and latitude 2° 23' north. The study involved collection of rectal swab samples from diarrheic and non-diarrheic suckling piglets and weaners (≤2 weeks after weaning) from smallholder herds for bacteriological analysis, and postmortem examination of very weak suckling piglets with severe diarrhea.

### Characteristics of pig herds in the study area

The majority of the study pig herds in northern and eastern Uganda were previously identified as smallholder each on average with 3 adult pigs, 7 to 8 suckling piglets, 5 weaners, 2 to 3 growing pigs with average herd size of 11 pigs [32]. The majority of the smallholder herds were comprised of local breeds of pigs and the most common method of management was tethering whereby the adults, weaners and growers were tied to the pegs with ropes and the suckling piglets let loose. Therefore, there is no housing of pigs in this system of management. It was common to find suckling piglets as old as 8 weeks hence weaned late. Diarrhea was a common major sign of disease especially in suckling piglets and weaners as reported by the pig owners.

### Collection and transportation of rectal swabs

Rectal swabs were collected from 32 diarrheic suckling piglets and weaners in smallholder herds with at least one diarrheic suckling piglet or weaner. Rectal swabs were also collected from 51 non-diarrheic suckling piglets and weaners in the same herds, and transported to the laboratory as previously described [33].

### Bacteriological culture, isolation and confirmation

The bacteriological cultivation for *E. coli* was performed in accordance with standard procedures [34]. Briefly, each rectal swab was directly cultured on sterile MacConkey agar (Mast group Ltd, Merseyside, UK) and incubated at 37 °C for 24 h. Four lactose-fermenting colonies from each sample were separately sub-cultured and biochemically confirmed using tryptophan broth for indole test, methyl red for MVP test and citrate agar for citrate utilization test. The biochemically confirmed *E. coli* (indole positive, MR positive, VP negative and citrate negative) were stored in brain heart infusion broth (Mast group Ltd, Merseyside, UK) with 20 % glycerol at - 20 °C until needed for DNA extraction.

### Determining hemolytic activity of *E. coli*

The *E. coli* isolates from diarrheic piglets and weaners were further cultured on blood agar containing 5 % horse blood (National Veterinary Institute, NVI, Uppsala, Sweden) and incubated at 37 ° C for 24 h for determination of hemolysis. For quality control, the beta-hemolytic in-house *E. coli* strain, 853/67; O149 (NVI, Sweden) was used.

### Serotyping of the *E. coli* isolates

The *E. coli* isolates from diarrheic piglets and weaners were inoculated in 2 mL of tryptic soy broth and incubated for 18 h at 37 °C followed by heating at 120 °C for 2 h to destroy the capsular antigen and release the O antigen. Then 100 μL of the boiled but cooled broth was mixed with 100 μL of the diluted O antisera in microtitre wells (with U-shaped bottom). The mixture was incubated overnight at 37 °C and the presence of agglutination was investigated the following day. Suspected agglutination was further tested by mixing 100 μL of the antigen with 100 μL of serially diluted antisera. The antisera used included the serogroups O6, O8, O9, O45, O46, O98, O101, O115, O138, O139, O140, O141, O147, O149, O157 and O179, provided by the National Veterinary Institute (NVI), Uppsala, Sweden.

### Post-mortem examination of piglets with severe diarrhea

Piglets that appeared weak and exhibited profuse diarrhea were clinically examined for other signs of disease before euthanasia [35]. Gross pathological lesions in the gastrointestinal tract were noted and tissue specimens from the duodenum, jejunum and ileum were collected and immediately fixed in 10 % buffered formalin. In the laboratory, the formalin-fixed tissues were processed, embedded in paraffin, sectioned and stained using hematoxylin and eosin following standard procedures [36]. Tissue sections were examined by light microscopy (400×, Axiostar Plus, Carl Zeiss MicroImaging GmbH, Gottingen, Germany) for histopathological lesions and photographed (Canon powershort A460, Canon Inc, China). The photos were then scanned using Zoom browser EX (Canon, USA) and saved in Microsoft office picture manager.

### Extraction of DNA from *E. coli*

In total, 83 frozen *E. coli* isolates, one isolate from each diarrheic and non-diarrheic pig were thawed and re-cultured on MacConkey agar at 37 °C for 24 h. From each isolate, DNA was extracted using the heat denaturation-rapid cooling on ice-centrifugation method [37, 38]. The extracted DNA was then aliquoted and kept at -20 °C until required for PCR amplification of sequences encoding the *E. coli* toxins and adhesins.

### The PCR amplification of gene sequences for F4, F5, F6, F18, F41, STa, STb, LT and EAST1

Two multiplex PCR (mPCR) sets were used to amplify the fragments of genes encoding the toxins and the fimbriae in one *E. coli* isolate from each pig. In the first PCR set, each reaction consisted of forward and reverse primers for STb, STa, LT, F6, and F4 (Table 1). In the second mPCR set, each reaction consisted of forward and reverse primers for EAST1, Stx2e, F41, F5 and F18 (Table 1). Each reaction consisted of 1× PCR buffer II, 3 mM MgCl_2_, 200 μM each of dATP, dTTP, dCTP and dGTP and 1.5 U of Ampli*Taq* Gold DNA polymerase (Applied Biosystems, Thermo Fisher Scientific Corporation, Massachusetts, USA). The cycling conditions for both mPCR sets were; 95 °C for 10 min, 35 cycles of 95 °C for 30 s, 59 °C for 30 s and 72 °C for 30 s followed by a final extension at 72 °C for 6 min. DNA from the in-house *E. coli* strains K88/NVI (F4^+^, LT^+^ and STb^+^), 853/67; O149 (F4^+^, F6^+^, LT^+^, STa^+^, STb^+^ and EAST1^+^), Bd 3437/83 I; O101 (F5^+^, F41^+^ and STa^+^) and Bd 60/84 I; O141(F18^+^, VT2e^+^, STa^+^ and STb^+^) (NVI, Uppsala, Sweden) and a blank sample without DNA were used as positive and negative controls, respectively.

**Table 1:** Primers used to amplify the fragments of the genes encoding the toxins and adhesins

Target	Primer sequence (5’ to 3’)	Conc. (pMol/μL)	Product size (bp)	Reference
STa	F: TTT CCC CTC TTT TAG TCA GTC AAC TG	0.05	160	[55]
	R: GGC AGG ATT ACA ACA AAG TTC ACA G	0.05		
STb	F: GCC TAT GCA TCT ACA CAA TCA	0.05	114	[55]
	R: TGC TCC AGC AGT ACC ATC TCT AAC CC	0.05		
LT	F: CCG GAT TGT CTT CTT GTA TGA	0.3	236	[56]
	R: TGT TCC TCT CGC GTG AT	0.3		
F6	F: TCT GCT CTT AAA GCT ACT GG	0.1	333	[57]
	R: AAC TCC ACC GTT TGT ATC AG	0.1		
F4	F: ATC GGT GGT AGT ATC ACT GC	0.5	601	[57]
	R: AAC CTG CGA CGT CAA CAA GA	0.5		
EAST1	F: TGC CAT CAA CAC AGT ATA TC	0.2	107	[56]
	R: GAG TGA CGG CTT TGT AGT C	0.2		
Stx2e	F: CCA GAA TGT CAG ATA ACT GGC GAC	0.1	322	[55]
	R: GCT GAG CAC TTT GTA ACA ATG GCT G	0.1		
F41	F: GCA TCA GCG GCA GTA TCT	0.2	380	[58]
	R: GTC CCT AGC TCA GTA TTA TCA CCT	0.2		
F5	F: TGG GAC TAC CAA TGC TTC TG	0.2	450	[57]
	R: TAT CCA CCA TTA GAC GGA GC	0.2		
F18	F: GTG AAA AGA CTA GTG TTT ATT TC	0.7	510	[59]
	R: CTT GTA AGT AAC CGC GTA AGC	0.7		
AIDA-I	F: TGCAAACATTAAGGGCTCG	0.05	450	[19]
	R: CCGGAAACATTGACCATACC	0.05		

### The PCR amplification of the gene sequence for AIDA-I

The *E. coli* isolates that tested positive for the toxin genes but negative for the genes encoding F4, F5, F6, F18 and F41 fimbriae, were tested for the presence of the gene encoding AIDA-I. Each PCR reaction consisted of 1× PCR buffer II, 3 mM MgCl_2_, 200 μM each of dATP, dTTP, dCTP and dGTP, 1.5 U of Ampli*Taq* Gold DNA polymerase (Applied Biosystems) and primers UN21 and UN22 (Table 1) that amplify a 450 bp fragment of AIDA-I. The cycling conditions were; 94 °C for 3 min, 35 cycles of 94°C for 30 s, 63 °C for 30 s and 72 °C for 30 s and a final extension step at 72 °C for 5 min.

### Agarose gel electrophoresis

Ten microliters of each of the PCR products were mixed with 2 μL of the loading buffer and resolved on 2 % agarose gel in 1× TBE buffer at 125 V for 45 min. The gel was stained by the SYBR® safe DNA gel stain (Life Technologies), imaged (Gel logic 200 imaging system, Kodak, New York, USA) and interpreted.

### Data analysis

Data on the *E. coli* virulence genes from diarrheic and non-diarrheic suckling piglets and weaners was coded and entered into SPSS version 17 (SPSS Inc., Chicago, USA). The data was analyzed using Chi-square test or Fisher’s exact test (when the requirements for Chi-square test were not met) for a difference in the prevalence of *E. coli* virulence genes from diarrheic and non-diarrheic suckling piglets and weaners.

## Results

### Number of *E. coli* and their sources

In total, *E. coli* isolates from 32 diarrheic suckling piglets and weaners, originating from 20 herds, were included. Of these, 11 suckling piglets were ≤ 1 month old and originating from 7 herds, 7 suckling piglets were > 1 month old and were from 6 herds, and 14 weaners originating from 7 herds. Piglets were generally weaned late, at least 2 months after birth. Weaning was abruptly performed mostly by removing the sow. In addition, *E. coli* isolates from 51 randomly selected non-diarrheic piglets and weaners from the same herds were tested.

### Post-mortem lesions in the diarrheic piglets

**Fig. 1:**
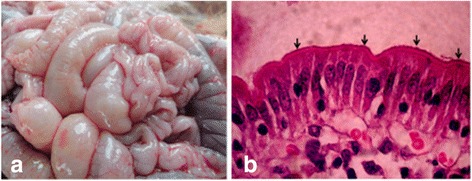
Post-mortem picture from an 8-week-old diarrheic piglet in northern Uganda. The segments of the jejunum were distended with fluid accumulation (**a**). Histopathology (**b**) showed intact jejunal villi, enterocytes and brush border but with cell infiltration of the jejunal epithelium. The bacteria (*black arrows*) can be seen attached to the brush border, forming a continuous layer. The *E. coli* strain AIDA/STb/EAST1, O139 was isolated from this piglet

Of the four diarrheic piglets examined, two piglets showed clinical and post-mortem pictures indicative of enterotoxigenic *E. coli* infection *i.e.* normal body temperature of 39.5 °C, distended small intestine with fluids (Fig. 1a), intact jejunal villi and enterocytes, and slight infiltration of inflammatory cells in the small intestinal epithelium (Fig. 1b). One 3-week-old piglet was emaciated and weak whereas the 8-week-old suckling piglet whose lesions are shown in Fig. 1a and b was stunted and had a rough hair coat. The DNA samples from these two piglets later tested positive for genes encoding *E. coli* virulence factors, EAST1 and AIDA/STb/EAST1, respectively.

### *E . coli* virulence factors detected from diarrheic and non-diarrheic piglets and weaners

**Fig. 2:**
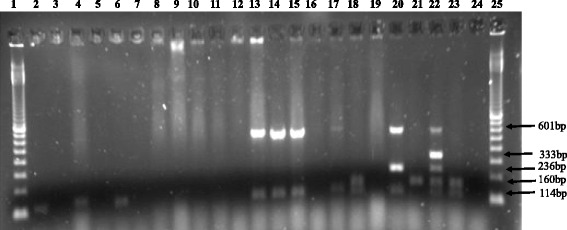
Electropherogram showing detection of virulence factors in *E. coli* isolates from diarrheic and non-diarrheic pigs. Lanes 1 and 25, 100 bp molecular weight marker (Bioron GmbH, Ludwigshafen, Germany). Lanes 2–4, 8–12,16–17, and 19 show *E. coli* DNA from the diarrheic pigs while lanes 5–7,13–15, and 18 show *E. coli* DNA from non-diarrheic pigs. Lanes 20, 21, 22 and 23, positive control DNA from *E. coli* isolates K88/NVI, Bd 3437/83 I, 853/67, and Bd 60/84 I, respectively. Lane 24, negative control consisting of a blank sample without DNA. The black arrows from top to bottom show the positions for F4 (601 bp), F6 (333 bp), LT (236 bp), STa (160 bp) and STb (114 bp). The PCR amplicons were electrophoresed on a 2% agarose gel stained with SYBR® safe DNA gel stain and visualized under UV-transillumination

**Fig. 3:**
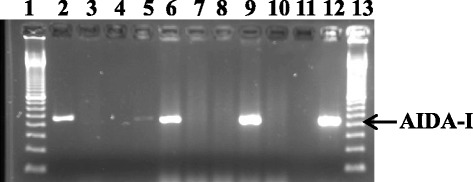
Electropherogram showing detection of AIDA-I gene in *E. coli* isolates from diarrheic and non-diarrheic pigs. Lanes 1 and 13, 100 bp molecular weight marker (Bioron GmbH, Ludwigshafen, Germany). Lanes 2, 6 and 7 are for the *E. coli* DNA from diarrheic pigs while lanes 3–5, and 8–10, are for *E. coli* DNA from non-diarrheic pigs. Lane 11, negative control consisting of a blank sample without DNA. Lane 12, positive control DNA from in-house *E. coli* isolate NVI024004 (AIDA-I^+^). Analysis shows 450 bp AIDA-I PCR products. The PCR amplicons were electrophoresed on a 2% agarose gel stained with SYBR® safe DNA gel stain and visualized under UV-transillumination

All the 83 *E. coli* isolates originating from 32 diarrheic and 51 non-diarrheic piglets and weaners were analysed for virulence factors (adhesin and toxin genes). Twenty-five fimbriae-negative but toxin-positive isolates originating from 25 pigs were analysed for AIDA-I. The genes encoding the *E. coli* toxins STa, STb and EAST1 were detected. The gene encoding LT was not detected in any of the 83 isolates examined (Fig. 2 and Table 2). The adhesin genes detected coded for F4 and AIDA-I while the genes encoding other adhesins (F5, F6, F18 and F41) were not detected (Figs. 2, 3, and Table 2). The prevalence of toxins, EAST1, STb, Stx2e, and STa, in the *E. coli* isolates from piglets and weaners (diarrheic and non-diarrheic combined) was 29, 26.5, 2.4, 1.2 %, respectively. The prevalence of the adhesins, AIDA-I, and F4 was 16, and 8.4 %, respectively. However, the prevalence of F4 and AIDA-I in *E. coli* from diarrheic piglets only was 22.2 and 20 %, respectively. There was no significant difference in the prevalence of the individual virulence factors in *E. coli* between the diarrheic and non-diarrheic pigs (*p* > 0.05). The ETEC strains identified from diarrheic and non-diarrheic pigs were those with only STb or EAST1 and those with virulence factor combinations including F4/STb/EAST1, F4/STb, AIDA/STb/EAST1, AIDA/STb, STb/STa/EAST1 and EAST1/Stx2e.

**Table 2:** Virulence factors (given both separately and in the various combinations) detected in *E. coli* isolates

Virulence factor(s)	No. with diarrhea [32]		No. without diarrhea [51]		No. positive	Prevalence %
	Suckling Piglets [18]	Weaners [14]	Suckling Piglets [44]	Weaners [7]		
STa	0	0	1	0	1	1.2
STb	7	2	10	3	22	26.5
EAST 1	6	2	12	4	24	29
Stx2e	0	1	0	1	2	2.4
LT	0	0	0	0	0	0
F4	4	0	3	0	7	8.4
F5	0	0	0	0	0	0
F6	0	0	0	0	0	0
F18	0	0	0	0	0	0
F41	0	0	0	0	0	0
AIDA	1 ^b^	1	1	1	4 ^a^	16
F4/STb/EAST1	3	0	3	0	6	7.2
F4/STb	1	0	0	0	1	1.2
AIDA/STb/EAST1	1	0	0	1	2 ^a^	8
AIDA/STb	0	1	1	0	2 ^a^	8
STb/EAST 1	0	1	2	2	5	6
EAST1/Stx2e	0	1	0	1	2	2.4
STb/STa/EAST1	0	0	1	0	1	1.2

One *E. coli* isolate was analysed from each pig

^a^ The number of AIDA-I-positive isolates originating from 25 isolates since only the fimbriae-negative but toxin-positive isolates were analysed

^b^ The number of AIDA-I-positive isolates from 5 isolates analysed from diarrheic piglets

### Serogroups and hemolytic activity of ETEC from diarrheic suckling piglets and weaners

From the 32 diarrheic suckling piglets and weaners originating from 20 herds, ETEC were isolated from seven piglets and two weaners from six herds. Isolates from seven suckling piglets and one weaner were serotyped. The isolates belonged to the serogroups O45, O138 and O139 and were non-hemolytic (Table 3 and Additional file 1). Where the serogroup could be determined, *E. coli* isolates from the same diarrheic pig were found to be of the same serogroup. Five of the diarrheic piglets were from semi-intensive systems while two piglets and one weaner were from tethering systems of management as previously defined [33].

**Table 3:** The serogroups and hemolytic activity of ETEC isolated from 7 diarrheic suckling piglets and 1 weaner

ETEC strain	Hemolysis	Serogroup	No. of pigs	Management method
F4/STb/EAST1	Non-hemolytic	O138	3	Semi-intensive
F4/STb	Non-hemolytic	None	1	Semi-intensive
AIDA/STb/EAST1	Non-hemolytic	O139	1	Tethering
AIDA/STb	Non-hemolytic	None	1	Tethering
F-/STb	Non-hemolytic	None	1	Semi-intensive
F-/STb	Non-hemolytic	O45	1	Tethering

## Discussion

This is the first study to identify ETEC as one of the etiologies of diarrhea in northern and eastern Uganda and to characterize their virulence factors. In Uganda, each smallholder farmer keeps on average 3 adult pigs and 8 piglets and diarrhea features as one of the major problems in piglets [32]. Hitherto, most of the information on ETEC diarrhea originates from countries where large-scale, intensive production system is predominant and weaning is performed at 3 to 5 weeks after birth [39]. In these systems, ETEC diarrhea is reported to be severe, highly prevalent and economically important [17, 40, 41]. However, findings in this study from parts of Africa where intensive system of production is less practiced, few pigs per household are kept [32] and weaning is done much later after birth, highlight that ETEC may be a problem in suckling piglets and weaners.

In the present study the most predominant adhesin detected in *E. coli* from diarrheic piglets was F4, in agreement with previous studies from developed countries [42–44]. Contrary to what has been commonly reported [3, 25], none of the F4/STb/EAST1-positive and F4/STb-positive ETEC strains from diarrheic piglets was hemolytic and most of them belonged to the O138 serogroup previously reported to be associated with diarrhea in piglets [45]. Reportedly, *E. coli* involved in PWD commonly belong to a few serogroups, including the O139 serogroup [46]. In addition, non-hemolytic F4-positive ETEC strains have also been detected in diarrheic piglets [42, 44] and the association between hemolysis and virulence is uncertain [8, 46]. Taken together, our data indicates that, virulent *E. coli* of varied serogroups circulate in pig herds from smallholder farmers in Uganda.

The F5, F6, and F41 adhesins were not detected in this study, suggesting that the prevalence of *E. coli* strains carrying these adhesins was very low. The F18 adhesin was also not detected. However, the F18 adhesin was recently reported in diarrheic weaners from large commercial farms in central Uganda [47]. Since F18 adhesin is associated with PWD [3, 48], this result could be due to the low number of diarrheic weaners tested. Secondly, the prevalence of PWD could be very low in weaners from smallholder herds, since this condition is mainly related to intensive rearing systems with high infectious load, abrupt changes in feeding regimes, stress caused by early weaning, and moving and mixing of animals. These conditions are usually not present in smallholder farming. However, ETEC diarrhea could be a problem in neonates from these smallholders since ETEC alone causes severe neonatal diarrhea with high mortality rates if left untreated [49].

The detection of AIDA-I in ETEC from a piglet with post-mortem findings strongly suggestive of colibacillosis continues to highlight the role played by this non-fimbrial adhesin in the pathogenesis of ETEC infection. It is not known whether the presence of the AIDA-I-positive strains in this study area has a zoonotic potential, since receptors for AIDA-I are also found on the human intestinal epithelial cells [50]. Thus, further studies are needed in this respect.

In agreement with previous studies that reported high prevalence of STb in *E. coli* isolates from suckling and weaned diarrheic cases, the most predominant toxin detected in *E. coli* from diarrheic piglets in this study was STb [3, 46, 51, 52]. The gene for EAST1 was the second most predominant detected from diarrheic piglets and this has also been previously reported to be highly prevalent among *E. coli* strains from diarrheic piglets [53]. In this study, the gene for LT was not detected and the gene for STa was detected in *E. coli* from one non-diarrheic piglet only, suggesting that the genes encoding for these two toxins are not widely spread. The absence of the gene for LT in all of the *E. coli*, more so in the STb-positive pathotypes, contradicts the results from a previous study [25] where a majority of STb-positive isolates were also LT-positive. In addition, the present study found the gene combination of STb/EAST1 in isolates from diarrheic piglets. However, since the role of EAST1 as a virulence factor is doubted [21, 29], and since the potent LT [7] is less prevalent, the ETEC diarrhea in this region could be largely contributed by STb in suckling and post-weaning pigs. The detection of Stx2e in weaned pigs suggests that the pigs are also at a risk of developing post weaning edema disease associated with this toxin.

All the 6 suckling piglets carrying the F4/STb/EAST1-positive *E. coli* were from the same household practicing semi-intensive piggery. This particular household had 2 adult pigs and 13 suckling piglets. Only 3 of these piglets had diarrhea at the time of sampling. Because of the cross-sectional study design, it is not known if the other, non-diarrheic piglets later developed diarrhea or were survivors that previously had experienced diarrhea. However, the high possibility of spread of the pathogen to all the piglets in such an enclosed system of management once one or a few piglets get infected was clearly demonstrated. Sick piglets will amplify the ETEC and the accumulation of fluids in the intestine will enhance excretion of the bacteria [54], thereby contaminating the environment.

## Conclusions

In conclusion, this study has identified ETEC in both diarrheic and non-diarrheic suckling piglets and weaners from the same smallholder herds. The ETEC strains carried two detectable adhesins and three toxins. The gross and histopathological findings suggest that piglets suffered from ETEC diarrhea and therefore, vaccination may be a suitable approach to control losses due to this diarrhea. However, more *E. coli* isolates and from different management systems in Uganda should be analysed so as to determine the most appropriate adhesin- based vaccines to use. There is also a need to investigate other causes of diarrhea *e.g.* viral infections since not all diarrheic pigs in this study were carrying ETEC.

## Abbreviations

AIDA, adhesin involved in diffuse adherence; CS, colonization surface antigen; EAST, *E. coli* aggregative heat stable toxin; ETEC, Enterotoxigenic *Escherichia coli*; F, fimbriae; LT, heat labile toxin; NVI, National Veterinary Institute; PCR, polymerase chain reaction; PWD, post-weaning diarrhea; ST, heat stable toxin; Stx, shiga-like toxin

## Additional file

Additional file 1: Diarrheic and non-diarrheic suckling piglets and weaners carrying *Escherichia coli* strains with virulence genes. (XLSX 13 kb)
